# Astrocytic Actions on Extrasynaptic Neuronal Currents

**DOI:** 10.3389/fncel.2015.00474

**Published:** 2015-12-09

**Authors:** Balázs Pál

**Affiliations:** Department of Physiology, Faculty of Medicine, University of DebrecenDebrecen, Hungary

**Keywords:** astrocyte-neuron interactions, slow inward current, slow outward current, tonic current, gliotransmitters

## Abstract

In the last few decades, knowledge about astrocytic functions has significantly increased. It was demonstrated that astrocytes are not passive elements of the central nervous system (CNS), but active partners of neurons. There is a growing body of knowledge about the calcium excitability of astrocytes, the actions of different gliotransmitters and their release mechanisms, as well as the participation of astrocytes in the regulation of synaptic functions and their contribution to synaptic plasticity. However, astrocytic functions are even more complex than being a partner of the “tripartite synapse,” as they can influence extrasynaptic neuronal currents either by releasing substances or regulating ambient neurotransmitter levels. Several types of currents or changes of membrane potential with different kinetics and via different mechanisms can be elicited by astrocytic activity. Astrocyte-dependent phasic or tonic, inward or outward currents were described in several brain areas. Such currents, together with the synaptic actions of astrocytes, can contribute to neuromodulatory mechanisms, neurosensory and -secretory processes, cortical oscillatory activity, memory, and learning or overall neuronal excitability. This mini-review is an attempt to give a brief summary of astrocyte-dependent extrasynaptic neuronal currents and their possible functional significance.

## Introduction

It was extensively demonstrated in the last decades that glial cells, especially astrocytes are not passive elements of the brain, but active partners of the neurons in signal processing (Araque et al., 1999, 2014; Perea et al., 2009). Astrocytes are known as non-excitable cells, as they can't produce action potentials. However, they can change their intracellular calcium concentration and produce calcium waves (Verkhratsky et al., 2012). This process (often termed “calcium excitability”) triggers gliotransmitter release. The best characterized gliotransmitters are glutamate, adenosine triphosphate (ATP), adenosine, γ-amino butyric acid (GABA), D-serine, and taurine. Astrocytes also release interleukin-1 (IL-1), tumor necrosis factor α (TNFα), neurotrophins, and prostaglandins (see Frank, 2013). Gliotransmitters are released by several mechanisms: either via calcium-dependent exocytosis (Parpura et al., 1994; Bezzi et al., 2004; Martineau, 2013), or transporters and channels such as cystine-glutamate antiport (Warr et al., 1999), connexon/pannexon hemichannels (Cotrina et al., 1998; Ye et al., 2003), ionotropic purinergic receptors (Duan et al., 2003), reverse mode of glutamate-, and GABA-transporters (Szatkowski et al., 1990; Gallo et al., 1991), volume-regulated anion channels and organic anion transporters (Rosenberg et al., 1994; Wang et al., 2002).

Astrocytes have complex functions in the central nervous system (CNS), including maintenance of the extracellular milieu for optimal neuronal function, regulation of synaptic plasticity, and participation in neuromodulatory actions. They contribute to sleep homeostasis, synchronization of neuronal networks, cortical oscillatory activity, chemosensitivity, and regulation of brain metabolism (Gourine et al., 2010; Halassa and Haydon, 2010; Henneberger et al., 2010; Poskanzer and Yuste, 2011; Navarrete et al., 2012; Frank, 2013; Lee et al., 2014). Astrocytes are affected in several pathophysiological processes such as neurodegenerative disorders, stroke, epilepsy, Alzheimer's, Huntington's and Parkinson's disease, amyotrophic lateral sclerosis, frontotemporal dementia, anxiety or primary cerebellar atrophy (Tian et al., 2005; Halassa and Haydon, 2010; Han et al., 2012; Shan et al., 2012; Orr et al., 2015; Sica, 2015; Zimmer et al., 2015).

As several aspects of astrocytic functions were thoroughly covered by numerous reviews, in this mini-review, I would like to focus on a distinct form of astrocyte-neuron communication, namely astrocytic actions on neuronal extrasynaptic currents.

## Neuronal extrasynaptic currents and their astrocytic control

Extrasynaptic currents can be detected in several areas of the CNS and show large heterogeneity in their kinetics, direction, neuro/gliotransmitters, and receptors responsible for them. These currents are elicited by ambient neurotransmitters acting on extrasynaptic neuro/gliotransmitter receptors. Ambient neurotransmitter levels are under control of both neurons and astrocytes, and have several potential sources such as spillover from synaptic clefts, neuronal volume transmission or somatodendritic release by neurons, and uptake or release of neuro/gliotransmitters by astrocytes (e.g., Semyanov et al., 2004; Okubo and Iino, 2011).

The following sections will focus on the astrocytic influences on extrasynaptic currents which will be grouped according to their time scale and direction.

### Slow inward currents: The “phasic” extrasynaptic action

Neuronal slow inward currents (SICs) are phasic extrasynaptic excitatory events distinguished from excitatory postsynaptic currents (EPSCs), due to differences in amplitude, rise time and decay time. The amplitude of these currents was 18–477 pA, with slow rise (13–332 ms) and decay times (72–1630 ms), fit by a single exponential function. In contrast, the amplitude of the miniature EPSCs was 19–40 pA, the rise time was significantly shorter (1–6 ms), whereas decay had a double exponential fit (τ_1_ = 6.6−27.6 ms, τ_2_ = 83−146 ms; Fellin et al., 2004; Shigetomi et al., 2008; Bardoni et al., 2010; Reyes-Haro et al., 2010).

**Figure 1:**
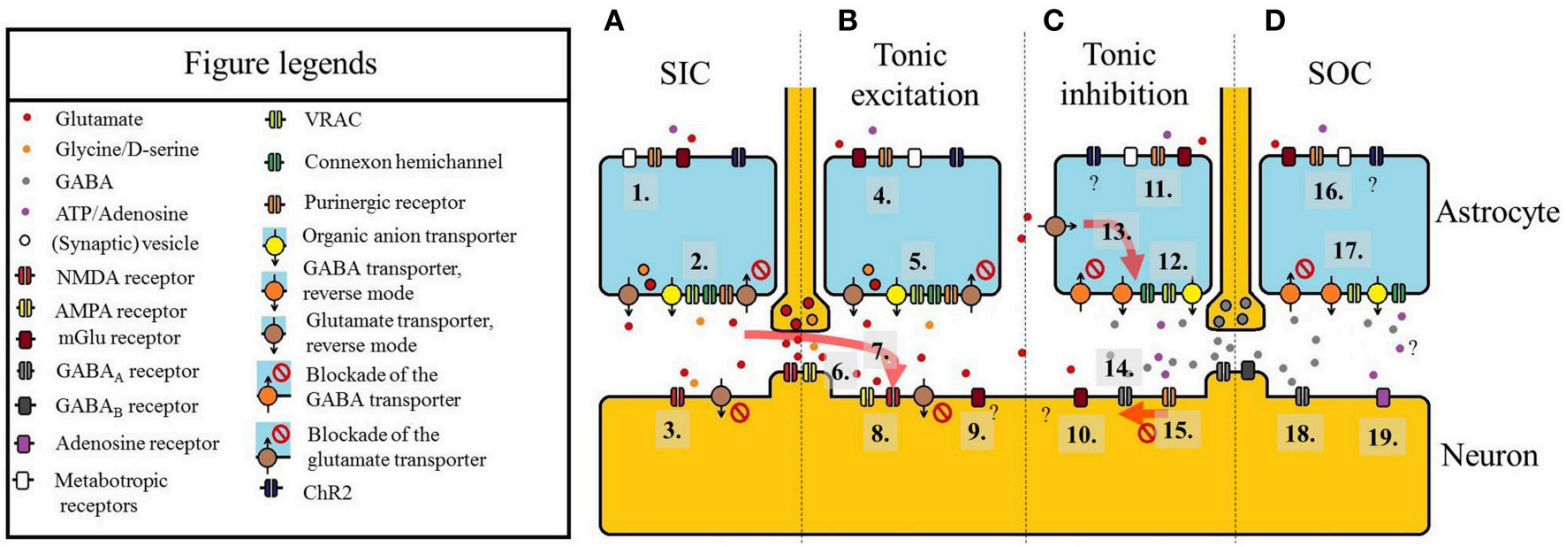
**Summary of the astrocytic actions on neuronal extrasynaptic currents**. **(A)** Slow inward current (SIC). (1) Astrocytes can be activated by purinoreceptors (e.g., P2X_7_), metabotropic glutamate receptors (mGluRs), other metabotropic receptors or channelrhodopsin-2 (ChR2) expressed in an astrocyte-specific way. (2) Glutamate (and D-serine) is released by astrocytes. Besides exocytosis of vesicles containing gliotransmitters, the reverse mode of glutamate transporters EAAT-1 and -2, organic anion transporters, volume-regulated anion channels (VRAC), connexon hemichannels, ionotropic purinergic receptors, or blockade of glutamate transporters are the molecules responsible for glutamate release. (3) Glutamate (and D-serine) diffuses to a neuron close to the release site and binds to extrasynaptic NMDA receptors. The activation of these receptors leads to SICs. Note that blockade of neuronal EAAT-2 can also contribute to the elevation of ambient glutamate levels eliciting SICs. **(B)** Tonic excitatory current. (4) Astrocytic activation via purinoreceptors (e.g., P2X_7_), mGluRs, other metabotropic receptors or channelrhodopsin-2 (see 1). (5) Release of glutamate and co-activators like D-serine (see 2). (6) Glutamate spillover from synaptic clefts (and volume transmission) also increases extrasynaptic glutamate concentration. (7) Diffusion of glutamate leading to SICs in a location closer to its release site can elicit tonic inward current. (8) Activation of extrasynaptic NMDA receptors, AMPA receptors and extrasynaptic mGluRs (9) lead to tonic excitatory currents. **(C)** Tonic inhibitory current. (10) Glutamate acting on a different set of mGluRs can elicit outward currents. (11) Activation of astrocytes by different receptors. Although astrocytes activated by ChR2 can potentially generate outward currents on certain neurons, it has not been directly shown. (12) GABA is released by GABA transporters in reverse mode (or by inhibition of the normal mode), VRACs or organic anion transporters. ATP is released via connexon hemichannels. (13) Increase of ambient glutamate concentration facilitates glutamate uptake and GABA release. (14) GABA released by astrocytes or resulted from volume transmission or spillover from synapses, activates extrasynaptic GABA_*A*_ receptors. (15) ATP activates P2X_4_ receptor which downregulates the number of GABA_*A*_ receptors, thus decreases the amplitude of tonic outward current. **(D)** Slow outward currents (SOCs). (16) Astrocytic activation (see 11). (17) Release of GABA and ATP/adenosine (see 12). (18) SOCs are generated by activation of extrasynaptic GABAA receptors. (19) SOC-like hyperpolarizing events can be seen by activation of A1 adenosine receptor. Adenosine stimulating these receptors likely has non-neuronal origin. A scheme of an excitatory synapse can be seen between panels **A** and **B**, whereas an inhibitory synapse is shown between panels **C** and **D**.

These currents are generated by activation of extrasynaptic NMDA receptors containing NR2B subunits, as SICs were prevented by general or NR2B subunit selective NMDA receptor antagonists. The involvement of NMDA receptors is also supported by the observation that appearance of SICs is largely facilitated in Mg^2+^-free extracellular solution. D-serine, a co-activator of the NMDA receptor also contributed to generation of SICs (Angulo et al., 2004; Fellin et al., 2004; Kozlov et al., 2006; D'Ascenso et al., 2007; Nie et al., 2010; Reyes-Haro et al., 2010; Pirttimaki et al., 2011; Pirttimaki and Parri, 2012; Figure 1).

SICs were found in several areas of the CNS, such as in the hippocampus (Angulo et al., 2004; Fellin et al., 2004; Perea and Araque, 2005; Carmignoto and Fellin, 2006), visual cortex (Chen et al., 2012; Perea et al., 2014), olfactory bulb (Kozlov et al., 2006), nucleus accumbens (D'Ascenso et al., 2007), thalamus (Parri et al., 2001), medial nucleus of the trapezoid body (Reyes-Haro et al., 2010), or the dorsal horn of the spinal cord (Bardoni et al., 2010; Nie et al., 2010).

It has been extensively demonstrated that SICs are consequences of astrocytic activity. Stimulation of astrocytes in astrocyte-neuron co-cultures led to the appearance of SICs on neurons in their neighborhood, and inhibition of astrocytic calcium signaling prevented the development of these events (Araque et al., 1998). When astrocytic intracellular calcium concentration was increased in slice preparations by Ca^2+^ uncaging, group I and II metabotropic glutamate receptor (mGluR) or muscarinic acetylcholine receptor agonists, ATP, prostaglandin E_2_ (PGE_2_), or optogenetic activation of astrocytes, the frequency of neuronal SICs was significantly increased (Angulo et al., 2004; Fellin et al., 2004; Perea and Araque, 2005; D'Ascenso et al., 2007; Bardoni et al., 2010; Pirttimaki et al., 2011; Chen et al., 2012; Perea et al., 2014). However, not all effects increasing astrocytic intracellular calcium concentration generated SICs on neurons: both P2Y_1_ and PAR-1 receptor stimulation on hippocampal astrocytes led to elevation of intracellular Ca^2+^, but only the latter one elicited SICs on pyramidal cells (Shigetomi et al., 2008). Blockade of action potential firing or release of synaptic vesicles did not affect the amplitude and frequency of SICs (Araque et al., 1998; Angulo et al., 2004; Fellin et al., 2004; Perea and Araque, 2005; D'Ascenso et al., 2007; Pirttimaki et al., 2011).

SICs recorded from different areas of the CNS have largely variable characteristics (Table 1) The large variance in their kinetic parameters implicate that gliotransmitters eliciting these events originate from nonsynaptic sources in a variable distance from their targets (Carmignoto and Fellin, 2006), and gliotransmitter concentration and the number of involved receptors shape the kinetics of SICs. Elevation of ambient glutamate concentration by the glutamate transporter inhibitor TBOA increased the amplitude of SICs (Angulo et al., 2004), and their rise and decay times became slower (Fellin et al., 2004).

**Table 1:** **Phasic excitatory and inhibitory currents of different brain areas**.

**Structure**	**Citation**	**Amplitude (pA)**	**Spontaneous frequency (1/min)**	**Rise time (ms)**	**Decay time (ms)**	**Triggered by (frequency increase)**	**Inhibited by (frequency decrease)**	**Synchronous activation of neighboring neurons**	**Manipulations eliciting SICs**
**PHASIC EXCITATORY CURRENTS (SLOW INWARD CURRENTS, SICS)**									
Nucleus accumbens	D'Ascenso et al., 2007	120.5 ± 9.3	0.05–0.2	81.4 ± 5.8	451 ± 42.2	DHPG, ATP, baclofen, low Ca^2+^ ACSF, uncaging Ca^2+^	D-AP5, ifenprodil	Yes	Stimulation of glutamatergic afferent; 10 trains with 30 Hz, repeated with 1 s intervals; MPEP prevented frequency increase elicited by stimulation
Olfactory bulb, granule cell	Kozlov et al., 2006	Approx. 30–100	0.24 ± 0.1				MK-801		
Ventrobasal thalamus	Pirttimaki et al., 2011; Pirttimaki and Parri, 2012	124.7 ± 0.5	0.07 ± 0.01	117.2 ± 20.8	831.4 ± 336.8	t-ACPD	D-AP5, ifenprodil		Lemniscal or cortical inputs; 10–20 stimuli with 50 Hz in every 5–10 s for 60–120 s. The frequency increase is prevented by group I. mGluR antagonists, and the effect remained for at least further 1 h.
Medial nucleus of the trapezoid body	Reyes-Haro et al., 2010	89.3 ± 9.7	0.275 ± 0.056		166.6 ± 16.3	Strychnine, gabazine, TTX after astrocyte stimulation	BAPTA dialysis of astrocytes, ifenprodil, MK-801+APV, DAAO	Rarely	Local electrical stimulation (amplitude of SICs increased)
Primary visual cortex	Chen et al., 2012; Perea et al., 2014	Approx. 26 and 15 pA	3.2 ± 1.1	13.08; 18.64 ± 2.31	116.47; 46.04 ± 3.42	Acetylcholine, photostimulation of ChR2-expressing astrocytes	BAPTA perfusion of astrocytes, D-AP5		
Hippocampus	Fellin et al., 2004	95 ± 36.7	0.16 ± 0.04	92.3 ± 29	538.5 ± 176	DHPG, uncaging Ca^2+^	D-AP5, ifenprodil, MK-801, 1 mM Mg^2+^	Yes	Stimulation of Schaffer collateral (100–200 ms long trains with 25–30 Hz frequency, 0.3–1 Hz repetition frequency)
	Angulo et al., 2004	104 ± 13	0.82 ± 0.15	135.5 ± 20	608.2 ± 216.45	DHPG, PGE_2_	D-AP5, MK-801	Yes	
	Perea and Araque, 2005	18.3 ± 1.4 (spontaneous); 77.7 ± 1.3 (evoked)	0.79 ± 0.09	13.9 ± 1.7	72.5 ± 11.1		D-AP-5, 2 mM Mg^2+^ *(the amplitude was unaffected by the latter one)*		Stimulation of Schaffer collateral
	Shigetomi et al., 2008	29 ± 1	0.03 ± 0.005	46 ± 18	196 ± 92	thrombin, TFLLR-NH_2_	BAPTA or fluoroacetate incubation, ifenprodil, D-AP5		
Spinal cord	Nie et al., 2010	477 ± 43.42	0.04 ± 0.003	332.46 ± 31.46	1630.61 ± 153.87	TBOA	TTX + TBOA; fluorocitrate + TBOA; D-AP5; Ro 25-6981 (amplitude reduction)		Stimulation of spinal dorsal root entry zone, in the presence of TBOA
	Bardoni et al., 2010	80.3 ± 12.8	0.01 ± 0.01 (12.5% of all tested neurons displayed SICs)	83.5 ± 16.1	423.1 ± 65.9	BzATP, low Ca^2+^			Peripherial inflammation (by intraplantar zymosan injection)
**PHASIC INHIBITORY CURRENTS (SLOW OUTWARD CURRENTS, SOCS)**									
Thalamic nuclei (dorsal lateral geniculate nucleus, nucleus reticularis thalami, ventrobasal complex)	Jiménez-González et al., 2011; Pirttimaki et al., 2013	111.3 ± 31.27	0.02 ± 0.01	108.9 ± 34.53		Hypo-osmotic stimulus, vigabatrin	Gabazine (SR95531)		
Olfactory bulb, mitral cell	Kozlov et al., 2006	266 ± 26	0.95 ± 0.16	47.2 ± 4.2	350 ± 25	Calcium-free ACSF, hypo-osmotic stimulus	Gabazine (SR95531), picrotoxin, bicuculline (partial)	Yes	Mechanical stimulation of astrocytes or blood vessels
Hippocampus	Le Meur et al., 2012	71.05 ± 14.99 (CA3) 46.95 ± 6.93 (DG)	0.04 ± 0.02 (CA1) 0.15 ± 0.04 (CA3) 0.34 ± 0.04 (DG)	35.7 ± 4.85 (CA3) 26.19 ± 8.41 (DG)		Hypo-osmotic stimulus	Gabazine (SR95531)		

*DHPG, mGluR I agonist; baclofen, GABA_B_ receptor agonist; D-AP5, NMDA receptor antagonist; Ifenprodil, NR2B-selective NMDA receptor antagonist; MPEP, mGluR5 antagonist; MK-801, NMDA receptor antagonist; t-ACPD, mGluR I-II agonist; Strychnine, glycine receptor antagonist; Gabazine (SR95531), selective GABA_A_ receptor antagonist; TTX, voltage-gated Na^+^ channel blocker; BAPTA, calcium chelator; DAAO, D-serine degrading enzyme; PGE_2_, prostaglandin E_2_; Thrombin, TFLLR-NH_2_, PAR-1 receptor agonists; Fluoroacetate, fluorocitrate, blockers of astrocyte metabolism; TBOA, glutamate transporter blocker; Ro 25-6981, selective NR2B receptor blocker; BzATP, purinergic receptor agonist; Vigabatrin, GABA transaminase inhibitor; Gabazine (SR95531), selective GABA_A_ receptor antagonist; Picrotoxin, bicuculline, GABA_A_ receptor inhibitors; DG, dentate gyrus*.

The functional role of SICs is likely the synchronization of neighboring neurons after longer excitatory stimulation by a significant input of their area. Longer stimulation of inputs increased the frequency of SICs in the nucleus accumbens, the hippocampus, and the ventrobasal thalamus. This synchronization was mostly demonstrated in forebrain structures: astrocytes can elicit SICs synchronously on pairs of neurons in the nucleus accumbens and on the hippocampal CA1 pyramidal neurons (Angulo et al., 2004; Fellin et al., 2004; D'Ascenso et al., 2007; Pirttimaki et al., 2011). In contrast, synchronization of SICs occurred rarely in the brainstem (Reyes-Haro et al., 2010).

SICs can be affected by a special form of plasticity: prolonged stimulation of afferents of the ventrobasal thalamus resulted a longer lasting (at least 60 min long) increase of SIC frequency, representing an astrocyte-dependent nonsynaptic plasticity (Pirttimaki et al., 2011).

### Slow outward currents: “Phasic” inhibition by astrocytes

Slow outward currents (SOCs) are rarely described phasic inhibitory events. Distinguishing them from neuronal inhibitory postsynaptic currents (IPSCs) is not as clear as in the case of SICs: although with their rise time of 26–109 ms and decay time of 350 ms they are significantly slower than GABA_A_ receptor-mediated fast IPSCs (3–8 ms decay time), their kinetic properties are closer to slow IPSCs mediated by GABA_A_ (30–70 ms decay time; Capogna and Pearce, 2011) and GABA_B_ receptors (80–100 ms rise time, 180–428 ms decay time; Degro et al., 2015).

GABA acting on (δ-subunit-containing) GABA_A_ receptors is responsible for generation of SOCs (Kozlov et al., 2006; Jiménez-González et al., 2011; Le Meur et al., 2012; Pirttimaki et al., 2013).

SOCs were recorded from the ventrobasal thalamus (Jiménez-González et al., 2011), the olfactory bulb (Kozlov et al., 2006), and from the hippocampus (Le Meur et al., 2012). SOCs can appear on the same neurons where SICs were also recorded: spontaneous inhibitory events had a significantly lower frequency than SICs in the ventrobasal thalamus and the hippocampal CA1 region, but SICs and SOCs had similar frequencies in the CA3 region and in the dentate gyrus (Jiménez-González et al., 2011; Le Meur et al., 2012).

The astrocytic origin of SOCs was demonstrated in the olfactory bulb, where mechanical stimulation of astrocytes or blood vessels was capable of eliciting these events (Kozlov et al., 2006). Contribution of astrocytes to SOCs was also shown by pharmacological blockade of neuronal activity and vesicle release, or facilitation of gliotransmitter release (Kozlov et al., 2006; Jiménez-González et al., 2011; Le Meur et al., 2012; Table 1; Figure 1).

Although not discussed as SOCs, astrocytic activity might raise other inhibitory events with slow kinetics. Rhythmical, hyperpolarizing, A_1_ adenosine-receptor mediated events were found in thalamic nuclei of the cat. The possible source of adenosine was the ATP released by non-neuronal structures (Lörincz et al., 2009).

The significance of the rarely identified SOCs is not known in great detail. However, they might have a role in neuronal synchronization, as they appeared synchronously on neurons of the olfactory bulb (Kozlov et al., 2006) and displayed reduced amplitude and slower kinetics in thalamocortical neurons from absence seizure model rats (Pirttimaki et al., 2013).

### Tonic excitatory currents regulated by astrocytes

Neuronal tonic excitatory currents were detected in several brain structures, but the relationship between these currents and astrocytic activity is less clear than with SICs. These currents are predominantly mediated by ambient glutamate and the NMDA receptor co-activator glycine (Le Meur et al., 2007; Papouin et al., 2012). Glutamate can originate both from neurotransmitter spillover and astrocytic release. Furthermore, ambient glutamate concentration can be decreased by uptake via different classes of glutamate transporters (Excitatory Amino Acid Transporters; EAAT1-4). EAAT1 and EAAT2 are predominantly glial, whereas EAAT3 and EAAT4 are neuronal (see Okubo and Iino, 2011; Zhou and Danbolt, 2013). Neuronal receptors of ambient glutamate are also heterogeneous: although extrasynaptic NMDA receptors contribute to the majority of tonic excitatory currents (Jabaudon et al., 1999; Angulo et al., 2004; Fellin et al., 2006; Le Meur et al., 2007; Fleming et al., 2011; Papouin et al., 2012; Petralia, 2012; Papouin and Oliet, 2014), involvement of AMPA receptors (Sasaki et al., 2012; Beppu et al., 2014) or group II mGluRs (Kõszeghy et al., 2015) was also demonstrated.

Tonic excitatory currents might appear together with SICs. The glutamate transporter inhibitor TBOA increased the amplitude of SICs together with activation of an NMDA-receptor dependent tonic current. It is likely that glutamate originating from the same astrocytic source elicited SICs on its closer targets, but activated a larger number of NMDA receptors with a lower concentration on distant activation sites on neurons, thus eliciting tonic inward current (Jabaudon et al., 1999; Angulo et al., 2004).

Tonic excitatory currents were detected in the hippocampus (Jabaudon et al., 1999; Angulo et al., 2004; Fellin et al., 2006; Le Meur et al., 2007; Papouin et al., 2012), the supraoptic nucleus (Fleming et al., 2011), the dorsal horn of the spinal cord (Nie et al., 2010), the pedunculopontine nucleus (Kõszeghy et al., 2015), and on cerebellar Purkinje cells (Sasaki et al., 2012).

Astrocytic contribution to tonic inward currents is supported by several indirect and direct observations. Inhibition of astrocytic functions by gliotoxins, EAAT1 and 2 glutamate transporters or blockade of astrocytic glutamine synthase increased the amplitude of the tonic inward current (Jabaudon et al., 1999; Angulo et al., 2004; Le Meur et al., 2007; Fleming et al., 2011). Stimulation of astrocytic P2X_7_ receptors or gliotransmitter release resulted the same effect (Fellin et al., 2004). As direct evidence for glial contribution, optogenetic stimulation of Bergmann glia elicited tonic inward neuronal current (Sasaki et al., 2012; Beppu et al., 2014). Optogenetic stimulation of astrocytes caused tonic depolarization and increased firing rate on neurons of the retrotrapezoid nucleus (Figueiredo et al., 2011) and the visual cortex (Perea et al., 2014). In these latter cases, the presence of tonic inward current was not demonstrated, but this current likely contributed to the observed phenomenon (Table 2, Figure 1).

**Table 2:** **Parameters of tonic excitatory and inhibitory currents in different brain areas**.

**Structure**	**Citation**	**Amplitude (pA), conductance (pS)**	**Time to peak (ms)**	**Elicited/potentiated by**	**Inhibited by**	**Relationship to SICs**
**TONIC EXCITATORY CURRENTS**						
Hippocampus	Fellin et al., 2006	−80 ± 23 pA	98 ± 9 s	BzATP (100 μM), potentiated by 0 Ca^2+^	D-AP5 (50–100 μM), OxATP (300 μM), BBG (2–4 μM)	Both were elicited by BzATP, but SICs were unaffected by OxATP or BBG
	Angulo et al., 2004	378.9 ± 87.8 pS (spontaneous, inhibited by D-AP5); 377.6 ± 54.9 pA (induced by TBOA, at +40 mV)		0 Mg^2+^, TBOA (100 μM)	D-AP5 (50 μM)	Triggered by overlapping mechanisms, appear together
	Jabaudon et al., 1999	331 ± 60 pA at +40 mV		TBOA (200 μM), MSO (1.5 mM)	D-AP5 (70 μM)	
	Le Meur et al., 2007	50.8 ± 13.4 pA at +40 mV		TBOA (100 μM), TBOA after preincubation with MSO	D-AP5 (50 μM), MK-801 (40 μM), 7-Cl-KYN (10 μM), PPDA (0.1 μM), NVP-AAM077 (0.1 μM)	
	Papouin et al., 2012	26.8 ± 3.1 pA at +40 mV (inhibited by D-AP5)			D-AP5 (50 μM), reduced by Ro25-6981 (2 μM) and BsGO	
Spinal dorsal horn	Nie et al., 2010,	−75.5±11.25 pA at −70 mV		TBOA (100 μM)		Both triggered by TBOA, appear together
Supraoptic nucleus	Fleming et al., 2011	31.8 ± 4.8 pA at +40 mV; −7.4±1.3 pA at −70 mV		Dihydrokainate (300 μM), TBOA (100 μM), α-AA (2 mM)	Kynurenic acid (2 mM), ifenprodil (10 μM), D-AP5 (100 μM), memantine (30 μM)	
Cerebellum, Purkinje-cells	Sasaki et al., 2012			Optogenetic stimulation of Bergmann glia; TBOA (100 μM)	GYKI 53655 (100 μM), NBQX (10 μM), DIDS (1 mM)	
	Beppu et al., 2014			Optogenetic stimulation of Bergmann glia; oxygen and glucose deprivation	GYKI 53655 (100 μM), DIDS (1 mM)	
Pedunculopontine nucleus	Kõszeghy et al., 2015	−24.5±4.4 pA at −60 mV		ACEA (5 μM)	Only tonic depolarization was investigated; thapsigargin (1 μM), LY 341495 (10 μM)	
Olfactory bulb (mitral, external tufted cells)	Belluzzi et al., 2004	Approx. −300 pA at −90 mV, +200 pA at −60 mV (10 mM taurine); biphasic at −60 mV	1.98 ± 0.23 s (10–90% rise time)	Taurine (2.5–10 mM), GABA (rapid decay; 200 μM)	Bicuculline (10 μM), picrotoxin (10 μM)	
**TONIC INHIBITORY CURRENTS**						
Hypothalamic paraventricular nucleus	Park et al., 2009	32.79 ± 5.04 pA (300 μM nipecotic acid)		Nipecotic acid (100, 300 μM); β-alanine (100 μM)	Bicuculline (20 μM; if elicited by nipecotic acid) bicuculline (20 μM) and strychnine (10 μM), if elicited by β-alanine	
		89.25 ± 22.66 pA (100 μM β-alanine)				
		Inward current with symmetrical Cl^−^ concentrations				
Cerebellar granule cells	Lee et al., 2010	35.7 ± 4.1 pA at -60 mV with symmetrical Cl^−^ concentrations, blocked with SR95531			SR95531 (10 μM); NPPB (50 μM), NFA, DIDS (100 μM)	
Neocortex	Lalo et al., 2014	39.9 ± 8.3 pA at −80 mV, with symmetrical Cl^−^ concentrations, blocked by bicuculline		Impairment of SNARE in astrocytes (dn-SNARE)	Bicuculline (50 μM), TFLLR (10 μM), TFLLR + PPADS (10 μM)	
Pedunculopontine nucleus	Kõszeghy et al., 2015	19 ± 1.9 pA at −60 mV		ACEA (5 μM)	Tonic hyperpolarization was blocked by MPEP (10 μM) + CPCCOEt (100 μM), thapsigargin (1 μM)	
Hypoglossal motoneurons	Gomeza et al., 2003	62.2 pA at −70 mV with symmetrical Cl^−^ concentrations		Disruption of GlyT1 gene	Strychnine (10 μM)	
Spinal cord, lamina X neurons	Bradaïa et al., 2004	−20 to −50 pA at −60 mV, with symmetrical Cl^−^ concentrations		ORG24598 (10 μM); also potentiated by ORG25543 (10 μM)	Strychnine (1 μM)	

*BzATP, purinergic receptor agonist; D-AP5, NMDA receptor antagonist; OxATP, P2X_7_ receptor antagonist; BBG, P2X_7_ receptor antagonist; TBOA, glutamate transporter blocker; MSO, glutamine synthase blocker; MK-801, NMDA receptor antagonist; 7-Cl-KYN, NMDA receptor antagonist; PPDA, NR2C-selective NMDA receptor antagonist; NVP-AAM077, NR2A-selective NMDA receptor antagonist; dihydrokainate, EAAT2 blocker; α-AA, gliotoxin; Kynurenic acid, ionotropic glutamate receptor antagonist; Ifenprodil, NR2B-selective NMDA receptor antagonist; Memantine, extrasynaptic NMDAR antagonist; Ro25-6981, selective NR2B receptor blocker, BsGO, recombinant wild type Bacillus subtilis glycine oxydase; NBQX, GYKI53655, AMPAR antagonists; DIDS, anion channel inhibitor; ACEA, CB1 receptor agonist; Thapsigargin, SERCA inhibitor; LY431495, mGluR II antagonist; bicuculline, GABA_A_ antagonist; Nipecotic acid, non-selective GAT inhibitor; **β**-alanine, GAT3 inhibitor; Strychnine, glycine receptor antagonist; Gabazine (SR95531), selective GABA_A_ receptor antagonist; NPPB, calcium-activated chloride channel blocker; NFA, DIDS, anion channel blockers; TFLLR, PAR-1 agonist; PPADS, ATP receptor antagonist; MPEP, mGluR5 antagonist; CPCCOEt, mGluR1 antagonist; thapsigargin, SERCA inhibitor; ORG24598, GlyT1 inhibitor; ORG25543, GlyT2 inhibitor*.

With their regulatory role on tonic neuronal excitatory currents, astrocytes participate in protection of neurons against hyperexcitability, are involved in the pathogenesis of seizures, contribute to long term synaptic plasticity and learning, and regulate neuromodulatory actions.

Administration of the glutamate transporter inhibitor TBOA generated seizures (Montiel et al., 2005), and selective astrocytic activation and consequential glutamate release triggered epileptiform discharges (Kang et al., 2005). Stimulation of astrocytic cannabinoid type 1 (CB1) receptors induced prolonged epileptic activity (Coiret et al., 2012), and conditional deletion of EAAT2 in astrocytes led to seizures (Petr et al., 2015).

In cooperation with neuronal processes, astrocytic regulation of these extrasynaptic currents contributes to learning and synaptic plasticity. Mice heterozygous for the EAAT2 gene (with moderate loss of EAAT2 protein) exhibited altered learning abilities compared to wild type animals (improvement in cue-based fear conditioning, but worse context-based fear conditioning; Kiryk et al., 2008). Long-term depression (but not long term potentiation) needed activation of both synaptic and extrasynaptic NMDA receptors (by glutamate and its co-agonists, glycine and D-serine; Papouin et al., 2012), demonstrating the role of neuronal and astrocytic cooperation in synaptic plasticity.

Tonic excitatory currents elicited with the contribution of astrocytic activity are neuromodulatory mechanisms. Tonic excitatory currents appeared after stimulation of astrocytic muscarinic acetylcholine receptors and induced acetylcholine-dependent cortical plasticity (Chen et al., 2012). Astrocyte- and mGluR-dependent tonic inward and outward currents in endocannabinoid signaling were also demonstrated in the pedunculopontine nucleus (Kõszeghy et al., 2015).

### Tonic inhibitory currents by astrocytes

Neuronal tonic inhibitory currents mainly originate from activation of extrasynaptic GABA_A_ receptors (reviewed in: Semyanov et al., 2004; Cellot and Cherubini, 2013; Connelly et al., 2013; Lee and Maguire, 2014). Briefly, tonic GABA-mediated currents are present in several brain areas (cortex, hippocampus, cerebellum, thalamus, striatum, and brainstem). The structure of receptors responsible for tonic GABA_A_ currents might differ from the ones responsible for IPSCs. Extrasynaptic receptors consist mostly of δ-subunits, but γ-subunits can also contribute to the receptor composition in certain cases.

Similar to tonic inward currents, tonic GABAergic currents are not exclusively of glial origin. Ambient GABA can originate from neurotransmitter spillover or volume transmission as well, although astrocytes can also release GABA as a gliotransmitter via different mechanisms (through bestrophin channels, Lee et al., 2010; reverse mode of GABA transporters GAT2 and GAT3, Angulo et al., 2008). Glutamate uptake by astrocytes was also coupled with GABA release (Héja et al., 2012).

Tonic GABA currents can be regulated by astrocytes via ATP release. ATP promoted the down-regulation of GABA_A_ receptors via activation of the P2X_4_ receptor, and thus attenuated the effects of extrasynaptic GABA in the neocortex (Lalo et al., 2014).

In contrast to astrocyte-dependent tonic excitation, it has not been shown that optogenetic activation of astrocytes elicits hyperpolarization of neurons. However, based on *in vivo* measurements of the firing rates, certain neurons of the visual cortex responded with decrease of their firing rate to optogenetic stimulation of astrocytes (Perea et al., 2014). Although this change of activity can be the consequence of actions on network activity, the finding is at least not against the possibility that astrocytic activation can cause neuronal hyperpolarization.

Tonic inhibitory currents can be elicited by neuro/gliotransmitters other than GABA. Tonic glycinergic currents were generated on spinal cord neurons by either blockade of glial glycine transporter (Bradaïa et al., 2004) or on hypoglossal motoneurons by disruption of the GLYT1 gene (Gomeza et al., 2003). Tonic hyperpolarization and tonic outward currents activated by CB1 receptor agonists on certain neurons of the pedunculopontine nucleus were successfully prevented by blockers of group I mGluRs (Kõszeghy et al., 2015; Table 2; Figure 1).

The tonic outward current has variable roles in regulation of neuronal functions, such as setting neuronal excitability, contribution to network oscillations, and has developmental functions like inhibition of cell proliferation and stimulation of cell migration (Semyanov et al., 2004; Park et al., 2009; Connelly et al., 2013; Lee and Maguire, 2014).

## Concluding remarks

Astrocyte-dependent neuronal extrasynaptic currents seem to be a general feature of the CNS. These phenomena are not always consequences of astrocytic actions on neurons, but often represent interplay between neuronal and astrocytic activity. The degree of astrocytic contribution is different: SICs have unambiguous astrocytic origin, but the astrocytic component of tonic inward and outward currents can't be always clearly separated.

The (patho)physiological roles of these currents are also heterogeneous and sometimes contradictory: tonic inward and outward currents are generally thought to contribute to synaptic plasticity and memory, although similar neuronal tonic inward currents can be observed in models of Alzheimer's disease (Talantova et al., 2013). Astrocytic functions can be responsible for neuroprotective mechanisms via regulation of neuronal excitability; but, under pathological conditions, such as stroke or epilepsy, astrocytic malfunction aggravates neuronal injury by causing neurotoxicity (Tian et al., 2005; Seifert and Steinhäuser, 2013; Beppu et al., 2014). Astrocyte-dependent tonic currents are also parts of neuromodulatory mechanisms, where both excitation and inhibition can be seen on neurons from the same neurochemical subgroup (Kõszeghy et al., 2015).

Furthermore, the function of the generally observed SICs is also not fully resolved. Appearing on neighboring neurons at the same time and being elicited by well-defined afferentation of the investigated brain area, they can synchronize neuronal activity, but their role in synaptic plasticity, to the best of my knowledge, has not been thoroughly investigated yet.

Taken together, neuronal extrasynaptic currents influenced by astrocytes contribute to several pathophysiological processes, but the uniform role of these phenomena and the significance of pure astrocytic mechanisms in eliciting them still remain an exciting field for further investigations.

## Author contributions

BP wrote the paper.

### Conflict of interest statement

The author declares that the research was conducted in the absence of any commercial or financial relationships that could be construed as a potential conflict of interest.

## Abbreviations

7-Cl-KYN: *7*-chlorokynurenate
α-AA: alpha-aminoadipic acid
ACEA: arachidonyl-2′-chloroethylamide (*N*-(2-chloroethyl)-5*Z*,8*Z*,11*Z*,14*Z*-eicosatetraenamide)
ACSF: artificial cerebrospinal fluid
ATP: adenosine triphosphate
BAPTA: 1,2-bis(o-aminophenoxy)ethane-N,N,N',N'-tetraacetic acid
BBG: Brilliant Blue G
BzATP: 2′(3′)-*O*-(4-benzoylbenzoyl)adenosine-5′-triphosphate
CB1 receptor: cannabinoid type 1 receptor
CNS: central nervous system
CPCCOEt: 7-(hydroxyimino)cyclopropa[*b*]chromen-1a-carboxylate ethyl ester
DAAO: D-amino acid oxidase
D-AP5: D-(-)-2-amino-5-phosphonopentanoic acid
DHPG: 3,5-dihydroxyphenylglycine
DIDS: 4,4′-diisothiocyanato-2,2′-stilbenedisulfonic acid
DG: dentate gyrus
EAAT1-4: excitatory amino acid transporter type 1-4
EPSC: excitatory postsynaptic current
GABA: *γ-amino butyric acid*
GAT2-3: GABA transporter type 2-3
GLYT1: glycine transporter type 1
GYKI53655: 1-(4-aminophenyl)-3-methylcarbamyl-4-methyl-3,4-dihydro-7,8-methylenedioxy-5*H*-2,3-benzodiazepine
Il-1: interleukin-1
IPSC: inhibitory postsynaptic current
LY341495: (2*S*)-2-amino-2-[(1*S*,2*S*)-2-carboxycycloprop-1-yl]-3-(xanth-9-yl) propanoic acid
mGluR: metabotropic glutamate receptor
MK801: (5*R*,10*S*)-(-)-5-methyl-10,11-dihydro-5*H*-dibenzo[*a,d*]cylcohepten-5,10-imine
MSO: methionine sulfoximine
NBQX: 2,3-dioxo-6-nitro-1,2,3,4-tetrahydrobenzo[*f*]quinoxaline-7-sulfonamide
NMDA: N-methyl-D-aspartate
NP-EGTA: *o*-nitrophenyl-ethylene glycol-*bis*(2-aminoethylether)-*N,N,N',N'-tetraacetic acid*
NPPB: 5-nitro-2-(3-phenylpropylamino)benzoic acid
NR2B: NMDA receptor subunit 2B
NVP-AAM077: [(S)-{[(1S)-1-(4-bromophenyl)ethyl]amino}(2,3-dioxo-1,2,3,4-tetrahydroquinoxalin-5-yl)methyl]phosphonic acid
ORG24598: *N*-methyl-*N*-[(3*R*)-3-phenyl-3-[4-(trifluoromethyl)phenoxy]propyl]-glycine
ORG25543: *N*-[[1-(dimethylamino)cyclopentyl]methyl]-3,5-dimethoxy-4-(phenylmethoxy)benzamide
OxATP: oxidized adenosine triphosphate
P2X_7_: purinergic receptor type 2X_7_
P2X_4_: purinergic receptor type 2X_4_
P2Y_1_: purinergic receptor type 2Y_1_
PAR-1: protease-activated receptor type 1
PGE2: prostaglandine E2
PPDA: (2*S*^*^,3*R*^*^)-1-(phenanthren-2-carbonyl)piperazine-2,3-dicarboxylic acid
Ro25-6981: (α*R*β*S*)-α-(4-hydroxyphenyl)-β-methyl-4-(phenylmethyl)-1-piperidinepropanol
SERCA: sarco/endoplasmic reticulum Ca^2+^-ATPase
SIC: slow inward current
SOC: slow outward current
t-ACPD: (±)-1-aminocyclopentane-*trans*-1,3-dicarboxylic acid
TBOA: *threo*-β-benzyloxyaspartic acid
TFLLR-NH2: Thr-Phe-Leu-Leu-Arg-NH_2_ (PAR1 agonist)
TNFα: tumor necrosis factor α
TTX: tetrodotoxin
VRAC: volume-regulated anionic channel.
